# Tuberculin—Some Personal Experience*Read before the Central Texas District Medical Society, January 9, 1917.

**Published:** 1917-03

**Authors:** W. O. Wilkes

**Affiliations:** Waco, Texas


### Tuberculin—Some Personal Experience.*

BY W. O. WILKES, M. D., WACO, TEXAS.

CASE 1.—Mrs. H., age 26, came to me for examination August 30, 1915. She had had a cough, slight fever and a little pain in the left shoulder for eight or ten months. Had been subject to "winter cough" for many years. Her mother died of tuberculosis.

*Read before the Central Texas District Medical Society, January 9, 1917.

In May her husband sent her to the mountains of Colorado, where she lived an open-air life for several months, but without improvement. Examination showed a tuberculous infiltration of the upper part of the left lung with slight involvement of the right apex. Her pulse at 11 a. m. was 87, temperature 98.2, systolic blood pressure 116, weight 128, and hemoglobin 75. She gave a history of .5 to 1.5 degrees of fever every evening. There were no tubercle bacilli in the sputum, but a tuberculin test was markedly positive. She was already living on a sleeping porch, and taking an abundance of wholesome food, so the only change made in her treatment was to give tuberculin subcutaneously. A general improvement was almost immediate and remarkable: appetite increased, and energy and vigor returned. In a very short time the pain and fever were entirely gone, and the cough practically nothing—in the morning only. The weight gradually went up to 143, a gain of fifteen pounds, and hemoglobin increased to 90, and in eight months she seemed to have a complete arrest of her disease, and was discharged. This patient had known what was the matter with her for a long time, and had tried everything that is usually recommended for tuberculosis except specific treatment, with no benefit. Cases of this kind, so-called incipient cases, show the best results from the use of tuberculin; cases that are practically at a standstill, or that are going down very slowly with little fever. Tuberculin will nearly always give these cases a boost in the right direction, and, with the other treatment usually recommended, will bring about recovery in practically all of them. There are scores, literally scores, of similar cases among my case records. Of course, some of them have had a recrudescence, and others may yet suffer a relapse, but, according to Lawrason Brown of the Adirondac Sanitarium, the percentage of relapses is 18 to 25 per cent greater in those who secure an arrest of their disease without tuberculin than in those whose diseases is arrested by using tuberculin with other treatment. It may be mentioned in passing that at the Adirondac Sanitarium patients are given their choice of using or not using tuberculin, and Lawrason Brown's figures were made by investigating the histories of patients who had been dismissed from the institution for a term of years.

Case 2.—H. L. S., architect, age 29, examined August 8, 1916. Has had slight cough, clearing of the throat and expectoration for three years. He suspected lung trouble and was examined by several physicians who told him his fears were groundless. His appetite was poor and he had lost 20 pounds. Examination showed a small destructive lesion in the upper part of the right lung, with some involvement of the left apex. His weight was 116, pulse, 114, temperature at 11 a. m. 99, systolic blood pressure 118, disatolic 70. Some tubercle bacilli were found in the sputum. Treatment without stopping him from his work was undertaken but had to be abandoned temporarily on account of gradually rising temperature, going finally to 101.5. He was put to bed on a sleeping porch and given respiratory infection mixed vaccine, and fed abundantly. Two weeks of this rest cure had his temperature so well under control that he could leave the bed for a time each day and begin graduated exercises. When he could stay up all day without his temperature rising to 100 in the evening tuberculin was begun, and this was after only one month of the rest treatment. He was not allowed to go to work, however, for two months. He is now, after five months' treatment, seemingly entirely well; has gained 30 pounds in weight, and is at work at his profession every day. There are quite a number of cases similar to this among my case records. Stopping from work and placing these moderately advanced cases on a sleeping porch, with rest and hygienic cure, usually gives all the stimulus of a change of climate.

Case 3.—Mrs. T. B., age 46, was first examined February, 1916. She had had advanced tuberculosis for some months. Her weight had gone down to 88 pounds, a loss of 38 to 40 pounds; systolic blood pressure 90, pulse 120 to 130, temperature 102 to 104 in the evening, expectorating large quantities of yellow sputum loaded with tubercle bacilli. Involvement of the larynx had affected her vocal cords so that she had been able only to whisper for several months. Night sweats were frequent and heavy. Apparently a hopeless case, and I so thought it. She was placed in bed on a sleeping porch and not allowed to sit up for any purpose, and forced feeding was instituted—all that her rather good digestive organs could take care of. A bitter tonic internally and mixed infection vaccine were the only medicines given her for the first six months. She remained in bed continuously for six months before her fever was under control. During this time she was gradually gaining in weight, night sweats disappeared, cough and expectoration diminished, bacilli disappeared from her sputum, and by September she was able to be out of bed all day without raising her temperature to 100; and then tuberculin was begun. Her voice has gradually returned, though there is still some huskiness at times, and she has gained 46 pounds in weight and is leading a comfortable, active life. She still has some cough and may have for the rest of her life. There are very few of these cured far advanced cases among my records; in the first place, they just don't do it very often, and in the second place nearly all of them that come under my observation go away for climatic or institutional treatment, or, for one reason and another, quit the treatment before the time arrives for active tuberculinization to be helpful or even safe.

CASE 4.—E. B. W., of Whitney, came to me December 11, 1913. Age 59, weight 122, pulse 95, temperature 99.5, systolic blood pressure 104, hemoglobin 70. He has had fever for five months, cough for many years. Examination showed chronic fibroid phthisis of both lungs, with some moisture in the right, showing an active process. There was also a mitral regurgitant murmur of the heart. Tuberculin was given him for seven months with a gain in weight of 14 pounds, and of hemoglobin to 85, and a general feeling of vigor and physical well-being. No fever since the beginning of the treatment. He has been under observation for his heart ever since, though his lungs are in good condition.

CASE 5.—Mrs. T. C., age 40, came for examination November 27, 1915. She has had "winter cough" for many years, has been coughing violently for six weeks, no expectoration. She is a large, ruddy, fleshy, healthy looking woman, weighing 170 pounds. An X-ray picture showed enlarged bronchial glands and some shadows that seemed to be old tuberculous infiltrations. She gave a history of having to be out of school for a year at the age of 12 on account of cough and slight fever. A tuberculin test gave a triple plus positive reaction. A few doses of tuberculin relieved her entirely of the cough. I do not know if it has returned this winter, as I have not seen her for several months.

CASE 6.—Mrs. J. M. J. came to me in November, 1914, with an ulcer on the outer side of the thigh about one and a half inches long by half an inch wide. It looked glazed and indolent, with perpendicular edges going down through the skin. Had been treated by a doctor in Denison for several weeks with no improvement. I treated it by stimulation, cauterization, and with antiseptics, and every way I knew, including mixed specific treatment internally, but with no benefit until tuberculin was resorted to. Injections were made around the ulcer and it began to heal immediately, with strong local reactions, and in a few weeks was entirely well.

CASE 7.—Mrs. S., of West. Age 35, weight 139, pulse 67, temperature 99 to 99.5 in the afternoon, blood pressure 108, slight cough. A neurasthenic with hysterical tendency. Enlarged bronchial glands and slight infiltration of the right apex. She was treated with tuberculin subcutaneously with early and substantial improvement of all her symptoms, and, in the course of a year, the neurasthenic and hysterical tendency had practically disappeared and the patient had gained 18 pounds in weight.

It has been my observation that a large percentage of neurasthenics are subjects of chronic tuberculosis, which in many cases affects the hilus glands only; and in many cases no other cause for the neurasthenia can be found. All these cases are benefited, and many are entirely cured, by tuberculin treatment.

These are some of the different kinds of cases that I have treated with tuberculin with good effect, and I have never had cause to regret its use in any case.

In the last eight years I have had under my care about 300 cases of tuberculosis—to be exact, 308 cases. Of this number about 20 per cent have gone away sooner or later for climatic or sanatorial treatment; approximately another 10 per cent have moved away from Waco, or quit the treatment for one cause and another before treatment was completed. About 7 per cent of the whole number have died. Of those who have remained under treatment for one year, or until dismissed, about 90 per cent have been cured, or have secured an arrest of their disease, which is the same thing for all practical purposes.

The value of tuberculin in cases proper for its use is so strong in my mind that I would as soon think of doing without it in these cases as I would of doing without quinine in malaria or mercury in syphilis.

It is not my intention to enter into any scientific discussion of tuberculin in this paper, for it seems to me that the value of vaccine therapy has been thoroughly established, and tuberculin is the first and archetype of vaccines.

I do not think there are many observant persons left in the world who will deny the value of Sir Almroth E. Wright's work in the opsonic index and vaccine therapy. Wright says a vaccine is useful in stimulating phagocytosis and the formation of antibodies and antitoxins in the blood in actute and chronic local infections and in chronic generalized infections. In acute generalized infection with fever the fever is due to an excess of the bacteria or their toxins in the blood, and to introduce more of either would be futile if not actually harmful. So it is that while typhoid vaccine is a certain prophylactic, it is not recommended as a cure by any scientific observer within my knowledge. So it is that no text-book places any value on the use of pneumococcic vaccine in acute lobar pneumonia. So it is that no one except one who will step in where angels fear to tread will give tuberculin in acute progressive febrile tuberculosis. These cases are put to bed, with absolute rest and the best hygiene, until the fever is under control, and then graduated exercise is instituted, and tuberculin in minute doses can be begun. In cases that have recently had high temperature or hemorrhage the initial dose must be exceedingly small. The cases of tuberculosis that give most beautiful results are those cases with cough, emaciation, low blood pressure, and only slight evening fever, like case 1 cited above. In these the improvement is prompt and remarkable.

It has been well said that tuberculin is a dangerous remedy, one to be handled with care and the greatest respect, for, like most powerful remedies, it is capable of doing immeasurable harm. At the same time there are few remedial agents in the armamentarium of the physician which are capable of doing a greater service to suffering humanity. Any one who tries to cure bedridden cases or acute, rapidly progressing cases with it will likely bring disaster to a trusting patient, and disrespect upon a wonderful remedy.

I will close this paper with a few quotations from recognized authorities.

Drs. von Ruck, in their book on Immunization Against Tuberculosis, say (page IX): "The possibility of influencing the tuberculous, and also the associated pathological processes by suitable specific remedies, has been established firmly, and it may be condemned almost as much to withhold specific treatment from a tuberculous patient who might be benefited from it, as it is to treat a case of diphtheria without antitoxin."

Cochrane and Sprawson, in their Guide to the Use of Tuberculin, page 5, approvingly quote Pottenger as follows: "Experience has now so accumulated that tuberculin does do good that the practitioner should look to the best methods of making use of it and not as to whether tuberculin is of use or not; in other words, he should blame his method of use and not the tuberculin in case of failure."

Hamman and Wohlman, of Johns Hopkins, in their book, say (page 242): "Tuberculin is not a 'cure' of tuberculosis—no more than hygiene or rest or diet or climate, or any other favorable factor. And, in making this comparison, we have really stated what tuberculin is—not a 'cure,' but a favorable factor."

Bandelier and Roepke, in their text-book, page 116, say: "Tuberculin treatment is in no way in opposition to the usual climatic treatment. The general treatment of the patient must rather be necessarily joined with the tuberculin treatment, the latter being of the greatest service when full advantage of the hygienic and dietetic *régime* under medical supervision can be taken at the same time. We, therefore, consider this combination of the two treatments to be at present the most efficient means of combating pulmonary tuberculosis. When the combination of the two treatments is not possible, tuberculin by itself must be employed whenever it can be done. More will certainly be achieved with it than by any other method of treatment used by itself."

Edward L. Trureau, who has been called the father of the tuberculosis sanatorium in America, said not long before his death that the more he used tuberculin the more confidence he had in it.

I could produce many other strong commendations of tuberculin from good authorities, and any one of you, by searching the literature, might find an equal number from equally as good authorities condemning it, and we should arrive nowhere. Those who have used it most have most faith in it. I will conclude with a quotation, which I heartily endorse, taken from Pottenger's Manual of Tuberculin in Diagnosis and Treatment:

"Tuberculin as a therapeutic measure is so well established today that when a clinician fails to obtain results by its employment he must consider that there is some error in his technic or his observation rather than believe that the remedy is at fault. I realize that the opinion of the profession is not unanimous in its endorsement of tuberculin, but this is nothing unusual. The fact that many good observers, who have studied tuberculosis carefully and who are devoting their energies to its cure, have been able to obtain good results from its use is sufficient to establish its value. The fact that other observers, some of whom are equally interested in the cure of tuberculosis, have not been able to secure good results should be looked upon as a personal matter rather than an argument against the remedy." "Much of the misunderstanding which has come about with reference to tuberculin as a therapeutic measure has been due to the fact that men have undertaken to administer it who know nothing about the remedy and very little about the disease. They do not know what it is expected to do, and have blamed it for their own misjudgments and mistakes. Tuberculin is not a perfect remedy. It will not cure all cases of tuberculosis."

"Tuberculin is a specific in tuberculosis—that is, its action is such that it calls out a physiological response on the part of the immunizing mechanism of the body, and in that way aids in specifically overcoming tuberculosis. The particular indication for its application as a remedy is in early tuberculosis. While not contraindicated in advanced tuberculosis, yet it must not be expected to have the same action when hampered by fibrosis and impaired functions on the part of the various organs as it does in early tuberculosis."

